# Id1 suppresses anti-tumour immune responses and promotes tumour progression by impairing myeloid cell maturation

**DOI:** 10.1038/ncomms7840

**Published:** 2015-04-29

**Authors:** Marianna Papaspyridonos, Irina Matei, Yujie Huang, Maria do Rosario Andre, Helene Brazier-Mitouart, Janelle C. Waite, April S. Chan, Julie Kalter, Ilyssa Ramos, Qi Wu, Caitlin Williams, Jedd D. Wolchok, Paul B. Chapman, Hector Peinado, Niroshana Anandasabapathy, Allyson J. Ocean, Rosandra N. Kaplan, Jeffrey P. Greenfield, Jacqueline Bromberg, Dimitris Skokos, David Lyden

**Affiliations:** 1 Children's Cancer and Blood Foundation Laboratories and Departments of Pediatrics and Cell and Developmental Biology, Weill Cornell Medical College, 413 East 69th Street, New York City, New York 10021, USA; 2 Drukier Institute for Children's Health and Meyer Cancer Center, Weill Cornell Medical College, 413 East 69th Street, New York City, New York 10021, USA; 3 Department of Neurosurgery, Weill Cornell Medical College, 1300 York Avenue, New York City, New York 10065, USA; 4 Department of Genetics, Oncology and Human Toxicology, Faculdade de Ciência Médicas, Universidade Nova de Lisboa, Rua da Junqueira 100, 1349-008 Lisbon, Portugal; 5 Regeneron Pharmaceuticals, Tarrytown, New York 10591, USA; 6 Melanoma and Immunotherapy Service, Department of Medicine, Memorial Sloan Kettering Cancer Center, 1275 York Avenue, New York City, New York 10065, USA; 7 Ludwig Center for Cancer Immunotherapy, Memorial Sloan Kettering Cancer Center, 1275 York Avenue, New York City, New York 10065, USA; 8 Department of Medicine, Memorial Sloan Kettering Cancer Center, 1275 York Avenue, New York City, New York 10065, USA; 9 Tumor Metastasis Laboratory, Fundación Centro Nacional de Investigaciones Oncológicas, Calle Melchor Fernández Almagro 3, 28029 Madrid, Spain; 10 Brigham and Women's Hospital, Department of Dermatology, Harvard Medical School, 221 Longwood Avenue EBRC, Room 513, Boston, Massachusetts 02118, USA; 11 Department of Medicine, Weill Cornell Medical College and Medical Oncology/Solid Tumor Program, 1305 York Avenue, New York City, New York 10021, USA; 12 Center for Cancer Research, Pediatric Oncology Branch, National Cancer Institute, National Institutes of Health, Building 10—Hatfield CRC, Room 1-3940, Bethesda, Maryland 20892, USA; 13 Department of Pediatrics, Memorial Sloan Kettering Cancer Center, 1275 York Avenue, New York City, New York 10065, USA

## Abstract

A central mechanism of tumour progression and metastasis involves the generation of an immunosuppressive ‘macroenvironment' mediated in part through tumour-secreted factors. Here we demonstrate that upregulation of the Inhibitor of Differentiation 1 (Id1), in response to tumour-derived factors, such as TGFβ, is responsible for the switch from dendritic cell (DC) differentiation to myeloid-derived suppressor cell expansion during tumour progression. Genetic inactivation of Id1 largely corrects the myeloid imbalance, whereas Id1 overexpression in the absence of tumour-derived factors re-creates it. Id1 overexpression leads to systemic immunosuppression by downregulation of key molecules involved in DC differentiation and suppression of CD8 T-cell proliferation, thus promoting primary tumour growth and metastatic progression. Furthermore, advanced melanoma patients have increased plasma TGFβ levels and express higher levels of ID1 in myeloid peripheral blood cells. This study reveals a critical role for Id1 in suppressing the anti-tumour immune response during tumour progression and metastasis.

## Abstract (web summary)

Tumour progression is promoted by the generation of an immunosuppressive macroenvironment. Here, the authors demonstrate that the Inhibitor of Differentiation 1 promotes the switch from dendritic cell differentiation towards myeloid-derived suppressor cell expansion during tumour progression.

## Graphical abstract

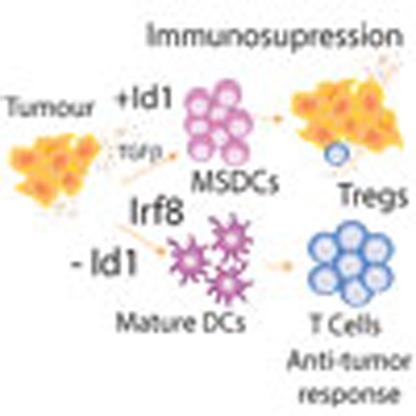

A pivotal mechanism of tumour outgrowth and progression to metastatic disease involves the ability of tumours to use a complex set of immunosuppressive mechanisms that prevent the immune system from mounting an efficient anti-tumour response^1^. Defective differentiation of bone marrow (BM)-derived myeloid cells (BMDCs) occurring in response to circulating tumour-derived factors is thought to lie at the core of this systemic tumour-induced immunosuppression^1^,^2^,^3^. Many tumour-derived factors, including vascular endothelial growth factor (VEGF), interleukin-4 (IL-4), IL-6, IL-13 and transforming growth factor beta (TGFβ), regulate redundant pathways likely related to myeloid cell differentiation^4^,^5^. In particular, these factors prevent the terminal differentiation of BMDCs into fully functional antigen-presenting cells (APCs), such as dendritic cells (DCs) and macrophages^6^,^7^. Instead, tumour-derived factors redirect myeloid differentiation towards the accumulation and expansion of a heterogeneous population of immature myeloid cells called myeloid-derived suppressor cells or MDSCs^1^,^8^,^9^.

DCs are the most potent APCs that are able to recognize, acquire, process and present antigens to naive, resting T cells for the induction of an antigen-specific immune response^10^. Increasing evidence shows that the main DC pathway affected in cancer patients is the myeloid DC pathway, particularly post chemotherapy^11^. The consequences of decreased numbers of functionally competent DCs in tumour-bearing hosts are clear: a decline in APCs renders immunostimulation less effective^6^,^7^. In contrast, an increase in MDSCs can have a profound immunosuppressive effects through T-cell suppression^3^,^5^,^12^,^13^.

MDSCs use a variety of antigen-specific and non-specific immunosuppressive mechanisms to suppress T-cell function, including increased arginase activity levels as well as nitric oxide and reactive oxygen species (ROS) production^14^,^15^,^16^,^17^. MDSCs have been found to accumulate in the circulation, lymphoid organs, primary and metastatic organs of most tumour models^18^, and in patients with various types of cancers including renal, breast and colorectal cancers^19^,^20^,^21^. MDSCs are thought to contribute towards the limited effectiveness of cancer vaccines and other therapies, such as anti-VEGF treatment^4^,^5^. However, it currently remains unknown whether tumour-secreted factors drive an alternative developmental pathway that co-regulates the decline in DCs and expansion of MDSCs via the upregulation of common transcriptional regulators during tumour progression.

The Inhibitor of Differentiation 1 (Id1) is a member of a family of transcriptional regulators that prevent basic helix–loop–helix transcription factors from binding DNA^22^,^23^. Increased Id1 protein expression in tumours has been shown to correlate with both cancer progression and poor prognosis^24^,^25^. Furthermore, Id1 regulates endothelial cell differentiation and fosters tumour vasculogenesis^26^,^27^, promotes progression from micro- to macrometastatic disease^28^ via endothelial progenitor cell mobilization and has been involved in myeloid development^29^,^30^,^31^,^32^. However, Id1 has not been previously involved in regulating the crosstalk between tumours and the host immune system at a systemic level and promoting tumour progression and metastasis via the suppression of myeloid cell differentiation.

In this study, we identify Id1 as a novel pivotal regulator of the switch from DC differentiation to MDSC expansion during tumour progression. We demonstrate that upregulation of Id1, primarily in response to tumour-derived TGFβ, redirects BMDC differentiation towards Id1-high expressing MDSCs with a reciprocal decrease in DC numbers. Id1 overexpression results in a systemic immunosuppressive phenotype that inhibits CD8 T-cell proliferation and increases primary tumour growth and metastatic progression. Our observations confirm and extend the promise of Id1 as a biomarker of cancer progression and as a therapeutic target in the management of advanced malignancies.

## Results

### Tumour-secreted factors favour *Id1*-high MDSC expansion

**Figure 1:**
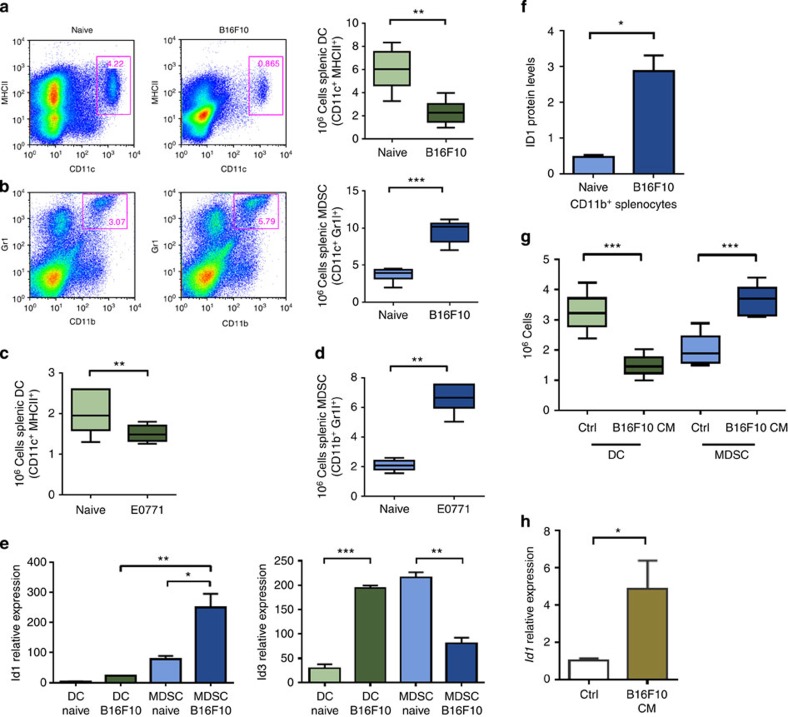
Tumour-secreted factors favour BMDC differentiation towards high Id1-expressing MDSCs but not DCs. Flow cytometry analysis of splenic populations from B16F10 melanoma-implanted mice (day 21 post implantation). (**a**) Frequency and absolute numbers of DCs (unpaired *t*-test **P<0.01). (**b**) Frequency and absolute numbers of MDSCs (unpaired *t*-test, ***P<0.001). Flow cytometry analysis of spleens from E0771 mammary adenocarcinoma-implanted mice (day 21 post implantation) for (**c**) DC absolute numbers and (**d**) MDSC absolute numbers compared with control mice (unpaired *t*-test, **P<0.01. (**e**) Id1 and Id3 mRNA levels in FACS-sorted splenic DC and MDSC populations, as determined by qPCR analysis, (*n*=6, one-way analysis of variance (ANOVA), *P<0.05, **P<0.01, ***P<0.001). (**f**) Id1 protein levels in lysates from naive and B16F10-bearing CD11b^+^ bead-sorted splenocytes as determined by western blot and densitometric analyses (unpaired *t*-test *P<0.01). (**g**) *In vitro* differentiation of Lin^−^ haematopoietic progenitors isolated from C57BL/6 mice, cultured for 6 days in the presence of B16F10 melanoma TCM (25% v/v), and analysed for DC and MDSC content by flow cytometry (*n*=6, ANOVA, ***P<0.001). (**h**) Id1 mRNA relative expression levels of day 6 Lin^−^ cells differentiated in the presence B16F10-conditioned media compared with control media, as determined by qPCR analysis (means±s.e.m., *n*=6, unpaired *t*-test, *P<0.05).

To assess differences in myeloid cell differentiation during tumour progression, we used the syngeneic B16F10 melanoma tumour model. Twenty-one days following inoculation of C57BL/6 mice with B16F10 melanoma cells (at the advanced metastatic stage), spleens were harvested and splenocytes were analysed by flow cytometry. We observed a decrease in the frequency and absolute numbers of DCs, defined as CD11c^+^MHCII^+^ cells, in B16F10 melanoma-bearing mice compared with non-tumour-bearing mice (2.6-fold; Fig. 1a), with both CD8^+^ and CD8^−^ DCs being affected but preserved at equal ratios (Supplementary Fig. 1A). Conversely, we detected an increase in the frequency and absolute numbers of MDSCs, defined as CD11b^+^Gr1^+^ cells, in tumour-bearing mice compared with controls (2.5-fold; Fig. 1b). Similar findings were observed in mouse spleens isolated 21 days after orthotopic implantation with the mammary adenocarcinoma E0771 cell line (Fig. 1c,d and Supplementary Fig. 1B,C).

As Id1 and Id3 upregulation in BM cells had been previously implicated in tumour and metastatic progression^27^,^28^,^33^, we sought to examine whether either of these transcriptional regulators were differentially expressed in DC and MDSC populations in tumour- versus non-tumour-bearing mice. Splenic DCs and MDSCs were isolated using fluorescence-activated cell sorting (FACS), and *Id1* and *Id3* expression was assessed by quantitative real-time PCR (qPCR) analysis. We found that DCs isolated from non-tumour mice expressed very low to undetectable *Id1*, whereas *Id1* expression was higher in MDSCs from tumour-bearing mice compared with both control MDSCs (3.2-fold; Fig. 1e) and DCs from tumour-bearing mice (11.2-fold; Fig. 1e). Similarly to *Id1*, *Id3* expression was higher in DCs from tumour-bearing mice compared to DCs from control mice, however, *Id3* expression levels in MDSCs from tumour-bearing mice were significantly lower compared to MDSCs from non-tumour-bearing mice (Fig. 1e). We therefore focused our subsequent studies specifically on Id1.

We then assessed Id1 protein levels in lysates from CD11b^+^ bead-sorted splenocytes isolated from naive or B16F10-bearing mice. The western blot and densitometric analyses revealed a 6.1-fold Id1 upregulation at the protein level in B16F10-bearing CD11b^+^ splenocytes compared with controls (Fig. 1f and Supplementary Fig. 1D). We also sought to examine whether Id1 expression is associated with a particular MDSC subtype—monocytic or granulocytic. Assessment of Id1 mRNA expression levels in FACS-sorted monocytic and granulocytic MDSC populations from spleens of naive and B16F10-bearing mice on days 7, 14 and 21 following implantation showed that increased Id1 expression is associated with both monocytic and granulocytic subsets, with increased levels in the monocytic subset in the earlier phase of tumour growth and increased levels in the granulocytic subset in the advance metastatic stage (2.5-fold and 3.5-fold, respectively; Supplementary Fig. 1E).

Similar experiments were performed with DC and MDSC FACS-sorted splenic populations from the E0771 mammary adenocarcinoma model, and the *Id1* expression profile was comparable to the one observed in the B16F10 model (Supplementary Fig. 1F).

As BM precursors give rise to all mature immune cells present in secondary lymphoid organs *in vivo*, we developed an *in vitro* model that mimics this differentiation process (BMDC assay). To determine whether differences in myeloid differentiation were due to circulating tumour-secreted factors, lineage negative (Lin^−^) haematopoietic progenitors were isolated from the BM of C57BL/6 mice and cultured for 6 days in the presence of B16F10 melanoma tumour-conditioned media (TCM) or control media. Using flow cytometry on day 6 of culture, we observed a decrease in the absolute DC numbers that differentiated in the presence in TCM, compared with control media (2.2-fold; Fig. 1g). In contrast, an increase in absolute MDSC numbers was observed on day 6 of culture with TCM compared with control media (1.8-fold; Fig. 1g). Gene expression analysis after 6 days of *in vitro* differentiation in B16F10 TCM revealed that Id1 mRNA expression levels were significantly higher in wild-type (WT; Lin^−^) cells differentiated in the presence of B16F10 TCM compared with control media (4.9-fold; Fig. 1h). Experiments performed with E0771 TCM revealed a similar imbalance in DC versus MDSC frequencies concurrent with Id1 upregulation (Supplementary Fig. 1G).

### *Id1* gene deletion restores myeloid differentiation defects

To assess whether Id1 is a direct regulator of MDSC and DC differentiation during tumour progression, we performed a series of experiments using *Id1*^*−/−*^ mice. As *Id1*^*−/−*^ mice have well-documented tumour angiogenic defects and abnormal tumour growth^27^, we performed daily injections of B16F10 melanoma TCM and control media over 21 days to systemically supply an equal amount of tumour-derived factors in both *Id1*^*−/−*^ and WT control mice.

**Figure 2:**
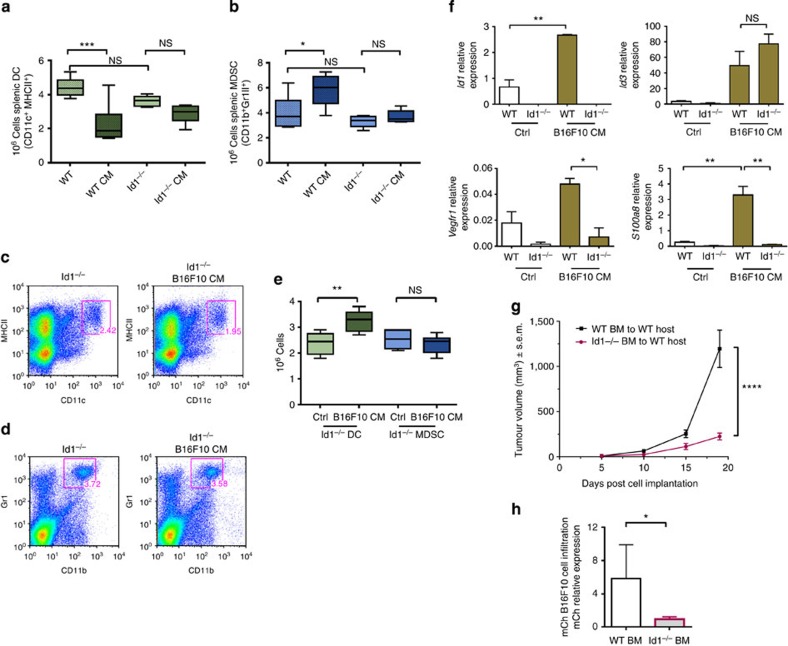
Deletion of the Id1 gene restores myeloid differentiation defects. Flow cytometry analysis of spleens from WT and *Id1*^*−/−*^ mice that received daily injections of B16F10 melanoma-derived conditioned media (B16F10 CM) or control media for (**a**) absolute numbers of DCs (one-way analysis of variance (ANOVA), ***P<0.001) and (**b**) absolute numbers of MDSC levels (one-way ANOVA, *P<0.05). (**c**) Representative frequency plots of DCs and (**d**) Splenic MDSCs isolated from *Id1*^*−/−*^ mice injected daily with B16F10 melanoma-derived TCM or control media. (**e**) *In vitro* differentiation of Lin^−^ haematopoietic progenitors isolated from *Id1*^*−/−*^ mice, cultured for 6 days in the presence of B16F10 melanoma TCM (25% v/v) and analysed for DC and MDSC content by flow cytometry (*n*=6, ANOVA, ^**^P<0.01). (**f**) Gene expression analysis of *Id1*^*−/−*^ and WT cells after 6 days of *in vitro* differentiation in the presence of TCM, as determined by qPCR analysis (means±s.e.m., *n*=6, one-way ANOVA, ^**^P<0.01, *P<0.05). (**g**) Analysis of primary tumour volume from *Id1*^*−/−*^ and control BM chimeric mice following implantation of B16F10 melanoma cells (two-way ANOVA, ****P<0.0001). (**h**) Relative quantification of mCherry-labelled B16F10 melanoma cells in cryosections of lungs of *Id1*^*−/−*^ and control BM chimeric mice measured by mCherry qPCR analysis (unpaired *t*-test, *P<0.05). NS, not significant.

B16F10 TCM injections led to a significant reduction in splenic DCs in WT versus TCM-treated WT mice (1.9-fold; Fig. 2a) that was comparable to the DC population reduction observed in tumour-bearing hosts. A non-statistically significant reduction in the DC population was detected in *Id1*^*−/−*^ mice injected with TCM versus control media (1.16-fold; Fig. 2a,c). Likewise, similar to the splenic MDSC expansion observed in tumour-bearing hosts, WT mice injected with TCM exhibited an increase in MDSCs compared with naive mice (1.5-fold; Fig. 2b), with both monocytic and granulocytic populations equally affected across groups (Supplementary Fig. 2A,B), whereas no expansion in MDSCs was seen with TCM injection in *Id1*^*−/−*^ mice (Fig. 2b,d).

In summary, genetic ablation of *Id1* largely restored terminal myeloid differentiation, as daily injections of B16F10 TCM prevented the DC reduction and MDSC expansion that was observed in WT controls that also received daily injections of B16F10 TCM. These data suggest that *Id1* has a critical role in mediating the myeloid differentiation defects caused by tumour-derived factors *in vivo* and support our previous findings in steady-state *Id1*^*−/−*^ mice where we observed an increase in terminal myeloid differentiation in the peripheral lymphoid organs and lower frequencies of common myeloid progenitors in the BM of *Id1*^*−/−*^ mice^23^. To further assess any impact of Id1 genetic deletion on DC progenitors, we also measured the frequency of the earlier common DC progenitor and myeloid DC progenitor populations in the BM of *Id1*^*−/−*^ and WT littermates. We found no difference in common DC progenitor frequencies between the two groups but a significant increase in the myeloid DC progenitor frequency in *Id1*^*−/−*^ mice, indicating an increase in dendritic myeloid differentiation with Id1 deletion (Supplementary Fig. 2C).

To further validate the role of Id1 in impairing myeloid differentiation in response to tumour-derived factors, we used the BMDC assay using *Id1*^*−/−*^ cells. In contrast to the results obtained with WT cells, we detected a significant increase in *Id1*^*−/−*^ DC numbers when BM progenitors were cultured in the presence of B16F10 melanoma TCM (1.4-fold; Fig. 2e) and no significant difference in MDSC numbers compared with controlled media cultures of *Id1*^*−/−*^ cells, indicating that Id1 has a causal role in the myeloid differentiation impairment observed in the presence of tumour-derived factors both *in vitro* and *in vivo*.

Gene expression analysis after 6 days of *in vitro* differentiation in B16F10 TCM revealed that the upregulation of *S100a8* and *Vegfr1*—two established markers of immature myeloid status—16,33,34,35 was abrogated in *Id1*^*−/−*^ Lin^−^ cells compared with WT cells (Fig. 2f). This suggested that in the absence of *Id1,* myeloid maturation is promoted. *Id3* expression levels were not found to be significantly different between WT and *Id1*^*−/−*^ cells cultured with TCM (Fig. 2f), excluding any potential compensatory mechanisms by Id3.

To further investigate the role of Id1 in primary tumour and metastatic progression, we transplanted Lin^−^ BM cells from *Id1*^*−/−*^ or WT BM into lethally irradiated WT recipients to generate BM chimeric mice. Eight weeks following BM transplantation, *Id1*^*−/−*^ and control BM chimeric mice were inoculated with mCherry-labelled B16F10 melanoma cells. Tumours from WT control chimeric mice showed a significant increase in volume compared with *Id1*^*−/−*^ BM chimeric mice at end point (day 19, 5.3-fold; Fig. 2g).

As B16F10 melanoma cells are known to metastasize to the lungs^33^,^35^, lungs from *Id1*^*−/−*^ and control BM chimeric mice were analysed for metastatic tumour burden by qPCR quantification of mCherry-labelled B16F10 melanoma cells. Lungs of WT control chimeric mice had a 6-fold increase in metastatic tumour cells compared with the lungs of *Id1*^*−/−*^ BM chimeric mice (Fig. 2h). These data further demonstrate a critical role for Id1-expressing BMDC in tumour and metastatic progression.

### *Id1* overexpression induces MDSC accumulation

To determine whether Id1 is indeed responsible for the development and accumulation of MDSCs in response to tumour-secreted factors, we transplanted lethally irradiated WT recipient mice with Lin^−^ BM cells from WT donor mice transduced with lentiviral vectors overexpressing *Id1* (OE Id1) or control vectors (ctrl). Both vectors also encoded for green fluorescent protein (GFP) to track transduced cells. Six to eight weeks after transplantation, the BM of recipient mice was reconstituted at a comparable reconstitution rate in both groups and over 90% of all cells in peripheral blood were positive for GFP.

**Figure 3:**
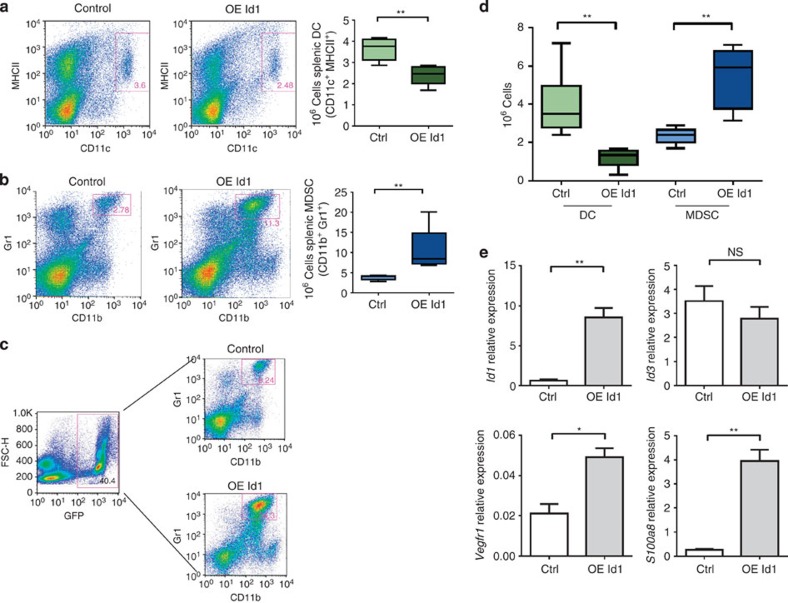
***Id1*** **overexpression induces a DC/MDSC imbalance.** Flow cytometry analysis of spleens from mice transplanted with Id1-overexpressing and control vector-transduced Lin^−^ BM cells for (**a**) frequency and absolute numbers of DCs and (**b**) MDSCs (unpaired *t*-test, ^**^P<0.01). (**c**) Representative percentages of MDSCs in GFP-positive splenocytes from mice transplanted with Id1-overexpressing Lin^−^ cells and control vector splenocytes. (**d**) *In vitro* differentiation of Lin^−^ haematopoietic progenitors from C57BL/6 mice transduced with lentiviral or control and Id1-overexpressing vectors overnight, cultured for 6 days and analysed for DC and MDSC content by flow cytometry (*n*=6, analysis of variance, ^**^P<0.01). (**e**) Gene expression analysis of Lin^−^ cells transduced with Id1-overexpressing and control vectors after 6 days of *in vitro* differentiation by qPCR analysis (means±s.e.m., *n*=6, unpaired *t*-test, ^**^P<0.01, *P<0.05; NS, not significant). Four independent experiments were performed.

Spleens from Id1-overexpressing and control vector mice were analysed 8 weeks post transplantation by flow cytometry for DC and MDSC levels. We found that, similar to defects seen in tumour-bearing mice, Id1-overexpressing mice exhibited a decrease in splenic DCs (1.5-fold; Fig. 3a) and an increase in MDSCs (2.7-fold; Fig. 3b) compared with control vector mice, with both granulocytic and monocytic populations being equally affected (Supplementary Fig. 3A,B). When assessing the percentage of MDSCs in GFP-positive splenocytes, we observed that 37.3% (±8.43%) of GFP-positive Id1-overexpressing cells were CD11b^+^Gr1^+^ compared with 8.14% (±2.43%) in GFP-positive control vector cells (Fig. 3c), confirming our hypothesis that Id1 expression favours BMDC differentiation towards MDSCs. These data also extend our previous observations in Id1-overexpressing mice, where we observed an increase in common myeloid progenitor frequency in the BM^23^.

To investigate the mechanisms by which Id1 impairs terminal myeloid differentiation, we performed the BMDC assay with WT cells transduced with lentiviral vectors overexpressing Id1 or control GFP only. After 6 days in culture, we observed a DC-MDSC imbalance similar to the one observed in cultures with TCM, with a significant increase in MDSC numbers at the expense of DC numbers in Id1-overexpressing mice (4.0 and 2.1-fold, respectively; Fig. 3d). Gene expression analysis of Lin^−^ cells transduced with Id1-overexpressing or control vectors after 6 days of *in vitro* differentiation showed a marked increase in *Vegfr1* and *S100a8* expression in Id1-overexpressing Lin^−^ cells compared with control cells (Fig. 3e), suggesting that Id1-overexpressing cells had a more immature phenotype than vector control cells.

### Id1-overexpressing MDSCs induce T-cell suppression

**Figure 4:**
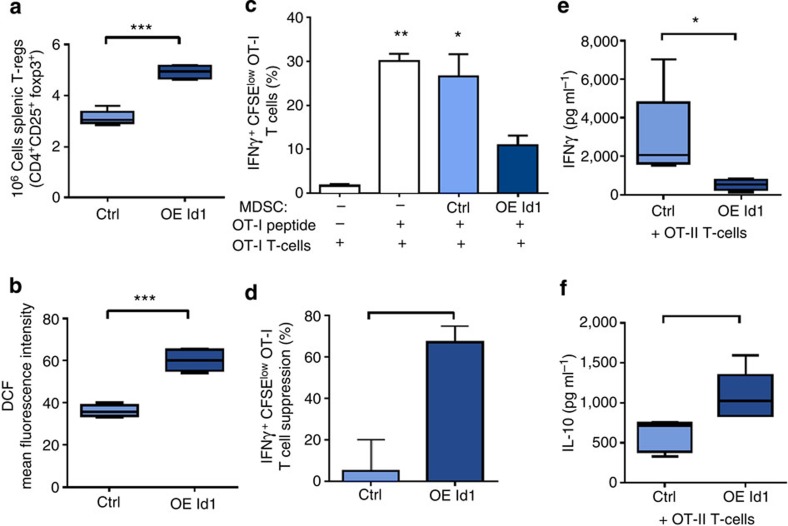
Id1 overexpression leads to an immunosuppressive phenotype and T-cell suppression. Flow cytometry analysis of spleens from mice transplanted with Id1-overexpressing and control vector-transduced Lin^−^ BM cells for (**a**) absolute numbers of regulatory T cells (T-regs; CD4^+^CD25^+^Foxp3^+^; unpaired *t*-test, ^***^P<0.001), for (**b**) ROS production, as determined by mean fluorescence intensity levels of dichlorofluorescein (DCF), a ROS-sensitive dye (unpaired *t*-test, ^***^P<0.001). (**c**) CD8^+^ antigen-specific T-cell proliferation functional assessment of GFP^+^ CD11b^+^ Gr1^+^ splenocytes from Id1-overexpressing and control vector animals co-cultured with OT-I splenocytes in the presence of OVA_257–264_ peptide. (analysis of variance, ^**^P<0.01, *P<0.05). (**d**) OT-I T-cell proliferation expressed as suppression induced by GFP^+^ CD11b^+^ Gr1^+^ splenocytes from Id1-overexpressing and control vector animals, relative to the no MDSC control wells. Data expressed as percentage T-cell suppression compared with no MDSC control (unpaired *t*-test, *P<0.05). (**e**) Analysis of splenocytes from Id1-overexpressing mice and OT-II CD4+ T-cell co-cultures in the presence of OVA_323–329_ peptide for IFNγ levels (unpaired *t*-test, *P<0.05) and (**f**) IL-10 levels compared with splenocytes from control vector-treated mice and OT-II CD4^+^ T-cell co-cultures (unpaired *t*-test, *P<0.05). Four independent experiments were performed.

To determine the consequences of BMDC Id1 overexpression on other measures of systemic immune function, we examined levels of regulatory T cells (T-regs), a group of highly immunosuppressive cells that have been previously described to expand in response to MDSCs^36^,^37^. Using flow cytometry analysis, we found that CD4^+^CD25^+^Foxp3^+^ T-reg absolute numbers were significantly increased among splenocytes of Id1-overexpressing compared with control vector mice (1.6-fold; Fig. 4a), supporting and extending our findings of an immunosuppressive role for Id1.

As MDSCs can exert their immunosuppressive effects via both antigen-specific and antigen-independent effects, we measured ROS production, thought to be one of the main non-antigen-specific MDSC-mediated immunosuppressive mechanisms^38^, in Id1-overexpressing splenocytes by flow cytometry. Measurements of fluorescence levels of dichlorofluorescein, a ROS-sensitive dye, indicated that splenocytes from Id1-overexpressing mice produce significantly higher levels of ROS than control vector splenocytes (1.7-fold; Fig. 4b), suggesting that non-antigen-specific mechanisms are also involved in the immunosuppressive phenotype that is generated by Id1 overexpression.

Next, we assessed antigen-specific immunosuppressive effects of Id1 overexpression on T-cell function using OVA antigen-specific T-cell co-culture models. Equal numbers of GFP^+^CD11b^+^Gr1^+^ cells isolated by FACS from Id1-overexpressing and control vector splenocytes were co-cultured in the presence of OVA_257–264_ peptide with Carboxyfluorescein succinimidyl ester (CFSE) stained OT-I splenocytes for 4 days. Quantification of proliferating (CFSE^low^) and activated (IFNγ^+^) CD8^+^ antigen-specific OT-I T cells showed a significant increase in T-cell proliferation in cultures with no CD11b^+^Gr1^+^ or control vector CD11b^+^Gr1^+^, but not with Id1-overexpressing CD11b^+^Gr1^+^ cells compared with control T-cell wells (no peptide; Fig. 4c). We observed a significant increase in T-cell suppression by Id1-overexpressing CD11b^+^Gr1^+^ cells compared with control vector (67.3 versus 5.3%; Fig. 4d). Furthermore, Th1/Th2 cytokine production analysis of conditioned media of splenocytes from Id1-overexpressing and control vector animals co-cultured in the presence of OVA_323–339_ peptide, and CD4^+^ OT-II cells showed a marked decrease in interferon-γ (IFNγ) levels (5.3-fold; Fig. 4e) and a significant increase in IL-10 levels (1.9-fold; Fig. 4f). Both assays indicate that Id1-overexpressing splenocytes and CD11b^+^Gr1^+^ cells, in particular, were able to directly suppress effector T-cell proliferation and activation, and promote a tolerogenic T-cell phenotype.

### Id1-overexpressing BMDCs promote tumour growth

**Figure 5:**
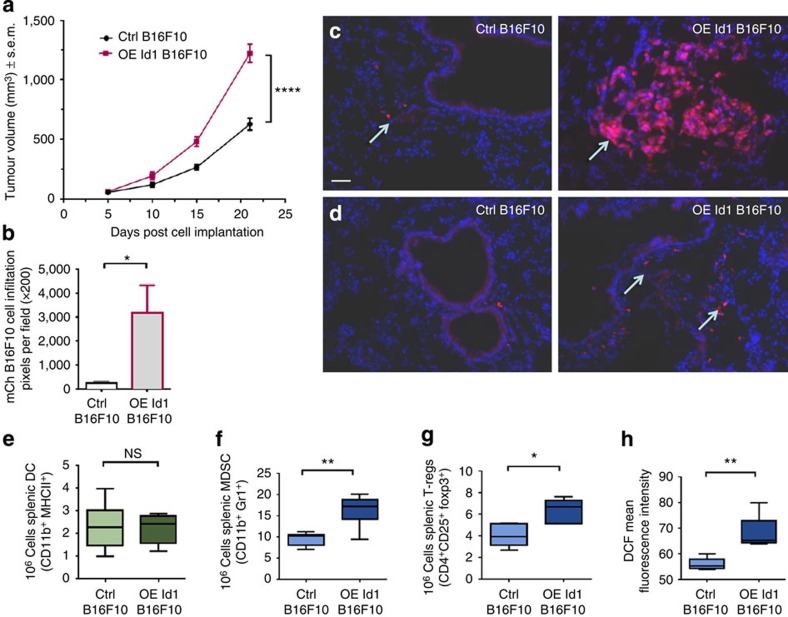
Id1-overexpressing BMDCs promote tumour growth and metastatic progression. (**a**) Analysis of primary tumour volume from Id1-overexpressing mice and control vector mice following implantation of B16F10 melanoma cells (two-way analysis of variance, ^****^P<0.0001). (**b**) Quantification of mCherry-labelled B16F10 melanoma cells in cryosections of lungs of BM Id1-overexpressing mice and control vector mice measured as red pixels per field (unpaired *t*-test, *P<0.05). (**c**) Macro- and (**d**) micrometastatic lesion formation in lungs from Id1-overexpressing mice and control vector mice; scale bar (50 μm) on top left panel applies to all panels. Flow cytometry analysis of splenocytes from BM Id1-overexpressing and control vector mice implanted with B16F10 melanoma cells for absolute numbers of (**e**) DCs (**f**) MDSCs (**g**) regulatory T cell (T-regs) and (**h**) ROS production (unpaired *t*-tests; NS, non-significant, *P<0.05, ^**^P<0.01). Four independent experiments were performed.

To determine whether the functional effects exerted by Id1 overexpression can alter tumour progression, 8 weeks following BM transplantation, Id1-overexpressing and control mice were inoculated with mCherry-labelled and non-labelled B16F10 melanoma cells. Tumour volume was measured during the model progression until day 21. Tumours from Id1-overexpressing mice had a significant increase in volume compared with control vector mice on day 21 (2.2-fold; Fig. 5a). Quantification of vessels by platelet/endothelial cell adhesion molecule-1 (PECAM-1^+^) staining and BMDC infiltration by GFP^+^ cell quantification on B16F10 tumour sections showed no statistically significant difference in vascularization or BMDC infiltration in the primary tumour of control mice compared with Id1-overexpressing mice implanted with B16F10 melanoma (Supplementary Fig. 4A,B).

Lungs from Id1-overexpressing and control vector-transplanted mice were analysed for metastatic tumour burden by quantification of mCherry-labelled B16F10 melanoma cells. Lungs of Id1-overexpressing mice had a 13-fold increase in metastatic tumour cells compared with the lungs of control vector-transplanted mice (Fig. 5b). Id1-overexpressing mice harboured significantly higher numbers of both micro- and macrometastatic lesions compared with vector-transplanted mice (Fig. 5c,d).

When we assessed the immune function of Id1-overexpressing tumour-bearing mice, we found similar DC numbers but significantly elevated MDSC (*P*<0.01), T-reg numbers (*P*<0.001) and ROS production (Fig. 5e–h respectively) compared with control vector tumour-bearing mice. These findings demonstrate that Id1 overexpression in haematopoietic cells is associated with an immunosuppressive phenotype and significantly increased primary tumour growth and metastatic burden.

### Id1 is upregulated via TGFβ and downregulates Irf8

To identify upstream regulators of Id1 and downstream pathways affected by *Id1* overexpression, we performed gene expression profiling of Id1-overexpressing and control BMDCs using Affymetrix GeneChip arrays. Microarray data are available in the ArrayExpress database (www.ebi.ac.uk/arrayexpress) under accession number E-MTAB-2280. Pathway analysis of the differentially expressed genes using Ingenuity Pathway Analysis software identified TGFβ and IL-6 among the top predicted upstream regulators of Id1 overexpression-induced gene expression changes (*P* value: 6.38 × 10^−29^ and 2.98 × 10^−21^, respectively).

**Figure 6:**
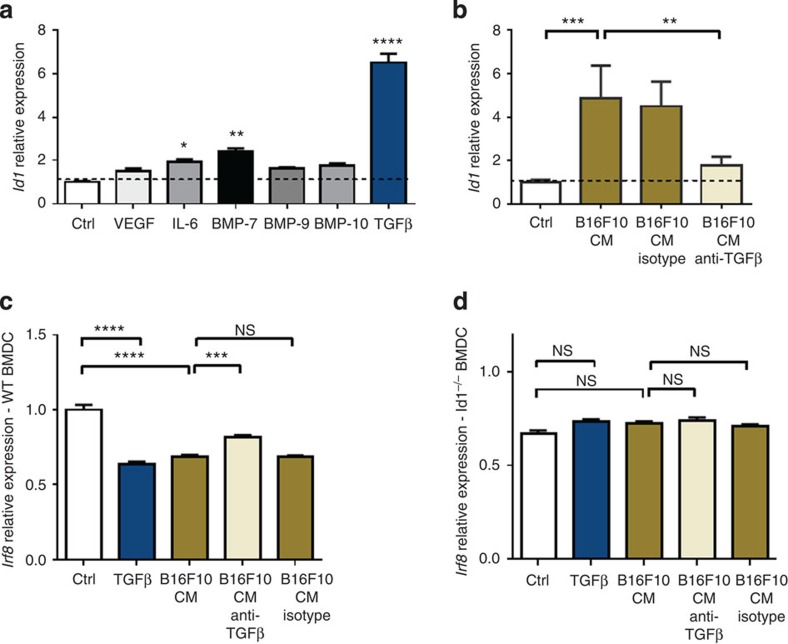
Id1 is upregulated via a TGFβ-dependent mechanism and downregulates key genes involved in DC maturation. (**a**) Id1 mRNA relative expression levels in day 6 Lin^−^ cells differentiated in the presence of 100 ng μl^−1^ of murine recombinant proteins (VEGF, IL-6, BMP-7, -9 and -10, and TGFβ compared with Lin^−^ cells differentiated in control media, as determined by qPCR analysis (means±s.e.m., *n*=6, analysis of variance (ANOVA), ^****^P<0.0001, ^**^P<0.01, *P<0.05). (**b**) Id1 mRNA expression levels in day 6 Lin^−^ cells differentiated in the presence of B16F10 CM (abbreviation introduced in Fig 2) alone, with anti-TGFβ and anti-IgG compared with control media, as determined by qPCR analysis (means±s.e.m., *n*=6, ANOVA, ^***^P<0.001, ^**^P<0.01). (**c**) Irf8 mRNA relative expression levels of day 6 WT Lin^−^ cells differentiated in the presence of 100 ng μl^−1^ of TGFβ, B16F10 CM alone, with anti-TGFβ and anti-IgG compared with control media, as determined by qPCR analysis (means±s.e.m., *n*=6, ANOVA, ****P<0.0001, ***P<0.001). (**d**) Irf8 mRNA relative expression levels of day 6 *Id1*^*−/−*^ Lin^−^ cells differentiated in the presence of 100 ng μl^−1^ of TGFβ, B16F10 CM alone, with anti-TGFβ and anti-IgG compared with control media, as determined by qPCR analysis (means±s.e.m., *n*=6, ANOVA; NS, not significant). Four independent experiments were performed.

To confirm that TGFβ and IL-6 were able to upregulate *Id1* in a relevant cell system, we tested these molecules as well as a series of candidate tumour-secreted factors previously implicated in MDSC expansion or Id1 upregulation^1^,^39^,^40^,^41^,^42^ in the BMDC assay. We observed that culture with TGFβ, and to a lesser extent IL-6 and Bone morphogenetic protein-7 (BMP-7), led to Id1 upregulation in BMDCs (6.5-, 1.9- and 2.4-fold respectively; Fig. 6a), confirming the two upstream pathway predictions of the microarray data analysis. *Id1* mRNA expression levels were found to be significantly higher in Lin^−^ cells differentiated in the presence of B16F10 TCM compared with control media (4.9-fold; Fig. 6b), whereas neutralization of TGFβ in B16F10 TCM largely prevented the upregulation of *Id1* by BMDCs (Fig. 6b). To determine whether these factors induce *Id1* expression in their soluble form or packaged in exosomes^43^, we quantified *Id1* expression in the presence of B16F10 soluble factors with and without exosomes, as well as B16F10 exosomes alone. We observed that *Id1* was upregulated by soluble factors but not exosomes (Supplementary Fig. 5A). We therefore concluded that soluble B16F10-derived factors induce *Id1* upregulation in MDSCs predominantly via a TGFβ-dependent mechanism.

Pathway analysis of the differentially expressed genes using IPA software identified the DC maturation pathway as one of the canonical pathways most significantly affected by Id1 overexpression (*P* value: 1.69 × 10^−3^; Supplementary Fig. 5B). Several key genes involved in DC maturation were found to be downregulated following *Id1* overexpression, including *Cd83*, *Cd86*, *MHCII (HLA-DQA1* and *HLA-DRB1)*, *Fscn1*, *Stat4* and *Irf8 (Icsbp)* (Supplementary Table 1).

Irf8 was of particular interest since it is a transcription factor that has been recently shown to restrict the generation of Gr1^+^ granulocytic populations such as neutrophils and MDSCs^44^, and to be responsible for initiating DC lineage commitment^45^. To further investigate the relationship between If8 and Id1 and determine whether Irf8 is a downstream mediator of Id1 function, we used the BMDC assay to assess the expression of *Irf8* in WT and *Id1*^*−/−*^ BMDCs in response to TGFβ and B16F10 TCM compared with control media. We observed a significant downregulation of *Irf8* expression in WT BMDCs in response to TGFβ and B16F10 TCM (1.6- and 1.5-fold respectively; Fig. 6c), an effect that was abrogated in *Id1*^−/−^ BMDCs treated with TGFβ or B16F10 TCM (Fig. 6d). Furthermore, neutralization of TGFβ in B16F10 TCM significantly reversed the downregulation of *Irf8* by BMDCs (Fig. 6c). We therefore concluded that B16F10 TCM induces *Irf8* downregulation in BMDCs via a TGFβ- and Id1-dependent mechanism. To identify the specific cell population that is primarily responsible for the TGFβ-mediated Id1 upregulation and Irf8 downregulation we observed in the BMDC assay and confirm whether these changes are occurring per cell or reflect overall cell population changes, we isolated DCs and MDSCs using FACS and assessed *Id1* and *Irf8* expression by qPCR analysis. We found that Lin^−^ cells cultured in the presence of recombinant TGFβ generate MDSCs expressing higher Id1 mRNA levels per cell (6.2-fold; Supplementary Fig. 5C) and DCs expressing lower Irf8 levels per cell compared with respective populations in control media cultures (5.9-fold; Supplementary Fig. 5D), confirming the inverse relationship of Id1 and Irf8 in specific isolated populations.

### Elevated ID1 levels in cancer patient CD11B^+^ PBMC

**Figure 7:**
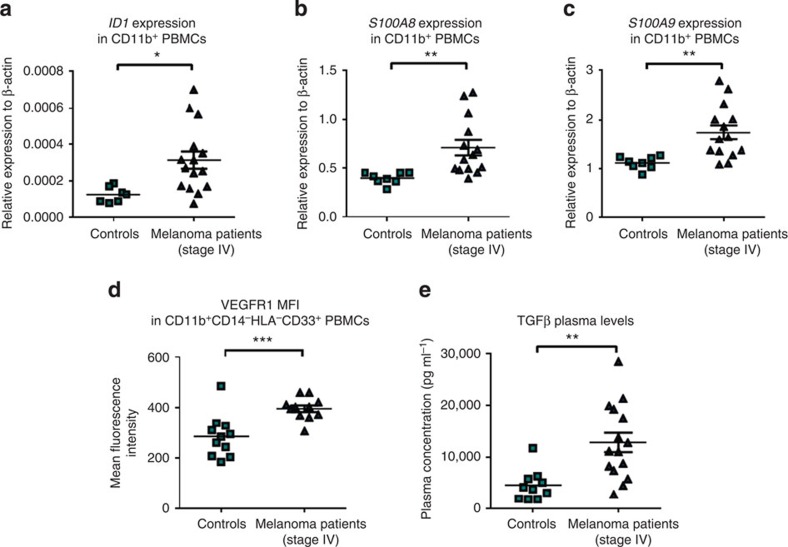
Advanced stage melanoma patients express higher levels of Id1 in the CD11B^+^ PBMC fraction and have elevated plasma TGFβ levels. (**a**) qPCR analysis of ID1 (unpaired *t*-test, *P<0.05), (**b**) *S100A8* (unpaired *t*-test, **P<0.01) and (**c**) *S100A9* (unpaired *t*-test, **P<0.01) mRNA expression levels following isolation of CD11B^+^ PBMCs from metastatic melanoma patient blood samples (*n*=15) compared with healthy matched controls (*n*=7). (**d**) VEGFR1 mean fluorescence intensity levels in CD11B^+^CD14^−^HLA^−^CD33^+^ cells PBMCs from metastatic melanoma patient blood samples compared with healthy matched controls, determined by flow cytometry (unpaired *t*-test, ***P<0.001). (**e**) TGFβ plasma levels from metastatic melanoma patients compared with controls, measured by ELISA (unpaired *t*-test, **P<0.01).

The frequency and numbers of MDSCs are increased in blood samples from most cancer patients including renal, breast and prostate cancer, as well as melanoma patients^46^,^47^,^48^,^49^. To establish the translational significance of our *in vivo* and *in vitro* findings, we measured the mRNA expression of *ID1* in CD11B^+^ cells isolated using magnetic beads from PBMC of patients with advanced melanoma (stage IV) and healthy age-matched controls. *ID1* expression was found to be on average 2.5-fold higher (and up to 5.4-fold higher) in CD11B^+^ PBMCs from patients with metastatic melanoma (*n*=15) compared with healthy, age-matched controls (*n*=7, unpaired *t*-test, *P*<0.05; Fig. 7a). Furthermore, *ID1*, but not *ID3* expression, was also found to be on average threefold higher (and up to ninefold higher) in PBMCs from patients with metastatic breast cancer (*n*=7) and colorectal cancer (*n*=6) compared with healthy, age-matched controls (*n*=10, Mann–Whitney test; *P*<0.01; Supplementary Fig. 6A,B).

Increased expression of the two downstream regulators of ID1, *S100A8 and S100A9* levels was also observed (1.8- and 1.7-fold higher and up to 3.1- and 2.7-fold, respectively) in CD11B^+^ PBMCs from patients with metastatic melanoma compared with healthy, age-matched controls (unpaired *t*-test, *P*<0.01; Fig. 7b,c). Flow cytometric analysis of VEGFR1 in the MDSC fraction of CD11B^+^ cells (defined as CD11B^+^ CD14^−^ HLA^−^ CD33^+^) also revealed elevated protein levels in stage IV melanoma patients compared with controls (unpaired *t*-test, *P*<0.001; Fig. 7d). Finally, we measured TGFβ levels in plasma samples from the same stage IV melanoma patients and observed a significant increase compared with controls (2.8-fold, unpaired *t*-test, *P*<0.01; Fig. 7e). Collectively, these data validate our pre-clinical observations in clinical samples of individuals with advanced malignancies.

## Discussion

Our study demonstrates a novel central role for Id1 in diverting normal myeloid cell differentiation from its intrinsic pathway of terminal differentiation to mature cells such as DCs towards a pathway that generates pathologically activated immature cells known as MDSCs^1^,^9^,^57^,^58^,^59^ during tumour progression. We demonstrate that Id1 upregulation is responsible for generating an immunosuppressive macroenvironment and driving tumour progression. We also demonstrate that Id1 overexpression specifically by MDSCs can directly suppress T-cell function. We identify TGFβ and IL-6 among the main tumour-derived factors responsible for Id1 upregulation in BMDCs, and demonstrate that Id1 and its upstream (TGFβ), as well as downstream mediators (S100A8/9 and VEGFR1) are significantly upregulated in advanced metastatic melanoma patients, confirming the translational value of our pre-clinical findings. In light of our results, we propose the use of Id1 and its mediators as biomarkers of systemic immune dysfunction during tumour progression as well as candidates for targeted anti-tumour therapeutic strategies.

Cancer is often considered to be a reflection of ‘embryonic memory'. *Id* genes are important in both embryonic neurogenesis and myocardial development^50^, and also regulate the self-renewal capacity of cancer-initiating cells^51^. *Id1* expression, in particular, correlates with both cancer progression and poor prognosis^24^,^25^. Prior studies have demonstrated a role for Id1 in endothelial cell differentiation and tumour vasculogenesis^26^,^27^, and progression from micro- to macrometastatic disease^28^ via endothelial progenitor cell mobilization. This is the first study to implicate Id1 in the crosstalk between tumours and the host immune system via regulation of myeloid cell differentiation.

Tumours release multiple factors that perturb the myeloid compartment^1^,^2^,^43^. These include VEGF, IL-4, IL-6, IL-10, IL-13, Macrophage colony-stimulating factor (M-CSF) and TGFβ, which regulate likely redundant pathways mediating the maturation and expansion of MDSCs at the expense of DC differentiation^1^,^4^,^13^,^52^ via transcription factors such as the signal transducer and activator of transcription 3 (STAT3) and CCAAT/enhancer-binding protein-α^13^,^53^. Hence, here we examined several tumour-derived factors that have been implicated either in MDSC expansion or Id1 upregulation^1^,^39^,^40^,^42^ in addition to factors that we identified as predicted upstream regulators of Id1-induced gene changes, and we identified TGFβ as one of the main tumour-derived factors responsible for Id1 upregulation in MDSCs. The link between TGFβ and Id1 appears to be context dependent^41^,^42^,^54^,^55^,^56^. Here, we show that in the case of BMDCs, TGFβ is the primary tumour-derived factor responsible for Id1 upregulation, as its neutralization largely abrogates Id1 expression *in vitro*. More importantly, we demonstrate that in melanoma patients, plasma levels of TGFβ and myeloid PBMC Id1 levels are both significantly upregulated.

Id1 has been shown to induce *S100a8/9* and *Vegfr1* expression, which have been previously associated with an immature myeloid phenotype. Specifically, the calcium-binding pro-inflammatory proteins S100A8 and S100A9 are thought to have key roles in myeloid differentiation and MDSC^57^ expansion, whereas VEGFR1 is a marker of immature myeloid cells^33^. These findings are also consistent with reports that VEGFR1^+^ cells may have impaired function in Id-mutant mice^27^ and that *Id1*^*−/−*^ DCs are not responsive to VEGF treatment via VEGFR1 (ref. 58). These findings support our previous observation that increases in VEGFR1 and Id expression occur in BMDCs and are largely responsible for driving the metastatic process^33^.

The transcriptional program driving MDSC development and expansion is poorly understood, partly due to the heterogeneity of MDSC subsets^18^,^59^. This study identifies Id1 as a new master transcriptional regulator of myeloid differentiation. Transcriptome analysis of Id1-overexpressing BMDCs revealed the downregulation of several genes thought to play a key role in DC maturation, such as those encoding the co-stimulatory molecules *Cd83* and *Cd86,* and *Irf8*, a transcription factor that controls DC lineage commitment^45^. Importantly, we demonstrate an inverse relationship and co-regulation between Id1 and Irf8. Humans with IRF8 mutations have a severe DC immunodeficiency syndrome^60^, whereas in murine studies Irf8 has been shown to impair the generation of Gr1^+^ granulocytic populations such as neutrophils and MDSCs, and to promote DC expansion and commitment^44^,^61^. Moreover, *Irf8* expression is decreased in MDSCs from tumour-bearing hosts and its overexpression leads to decreased MDSC levels^62^, suggesting that Irf8 is an important regulator of MDSC expansion during tumour progression. Our study provides novel insights into the molecular pathways that link the inhibition of DC maturation and MDSC expansion, identifying a previously unknown inverse relationship between Id1 and Irf8.

When examining the functional outcomes of systemic Id1-induced tumour immunosuppression, we identified both antigen non-specific and specific mechanisms by which Id1-expressing MDSCs exert their immunosuppressive effects. First, the increase in total ROS levels following Id1 overexpression comes in agreement with studies, demonstrating that ROS are major factors in the inhibition of DC differentiation and MDSC expansion in tumour-bearing mice^38^,^63^. As VEGFR1 expression is also thought to be regulated by oxidative stress^16^, these findings provide a mechanistic link between increased ROS and induced upregulation of VEGFR1, and identify Id1 as the molecular link between the two phenomena. Second, another key mechanism of MDSC-induced immunosuppression is the activation and expansion of T-regs^37^,^64^. Although these mechanisms are not completely understood, they are thought to involve cell-to-cell contact^65^ and the production of cytokines, such as IFNγ, IL-10 and TGFβ^64^. The significant decrease in IFNγ and increase in IL-10 detected in co-cultures of Id1-overexpressing splenocytes with naive OT-II CD4 T cells confirm the activation of antigen-specific immunosuppressive mechanisms. Finally, we demonstrate that Id1 overexpression in the CD11b^+^Gr1^+^ subset specifically induces antigen-specific T-cell suppression, providing direct evidence of the functional consequences of Id1 overexpression in downstream effector immune responses.

Despite well-documented evidence suggesting that malignant melanoma is an immunogenic tumour^66^, a property that has made this disease a preferred target for investigating different immunotherapeutic strategies^67^,^68^, clinical outcomes have not been as promising as anticipated. These seemingly paradoxical results are now thought to be due to an immunosuppressive environment generated by cells such as MDSCs^69^. Given the rise in incidence and death rates of metastatic melanoma^70^, there is increased urgency for a deeper understanding of the regulation of these pro-metastatic, immunosuppressive mechanisms.

Our study reveals for the first time a novel pivotal role for Id1 in tumour and metastatic progression and in controlling systemic tumour-induced immunosuppression, providing further insight into the therapeutic promise of Id1 targeting. Pharmacological inhibition of Id1 using blocking peptides or small interfering RNA would offer the advantage of selective targeting, therefore largely minimizing side effects. This new approach would offer the opportunity to re-examine immunotherapies in a new improved setting. Targeting of Id1 or downstream pathways would provide a three-pronged therapeutic approach by reducing metastatic potential of the tumour itself, reducing tumour angiogenesis and finally restoring systemic immune function.

## Methods

### Human studies

Human peripheral blood samples were obtained under informed consent and handled in accordance with approved Institutional Review Board protocols (IRB 0604008488 and IRB 12-137(A)). Human peripheral blood samples from stage IV melanoma patients at the Memorial Sloan Kettering Cancer Center had histologically confirmed melanoma. Plasma and PBMC were isolated as previously described^43^. CD11B^+^ cells were isolated by positive selection using CD11B-coated magnetic beads (Miltenyi Biotec).

### Mice

Generation of *Id1*^*−/−*^ mice has been previously reported^23^. Animals used in all experiments were matched for sex, age (8–10 weeks old) and genetic background (C57BL/6/Sv129). C57BL/6 mice were purchased from Harlan Laboratories or The Jackson Laboratory (Bar Harbor, ME); OT-II mice were obtained from The Jackson Laboratory. All animal procedures were approved and performed under the guidelines of the Institutional Animal Care and Use Committee (IACUC) at Weill Cornell Medical College, protocol (IACUC 0709-666A).

### Isolation and *in vitro* differentiation of Lin^−^ cells

BM cells were harvested from the femurs and tibias of 8–12-week-old mice and enriched for haematopoietic progenitor cells by depletion of lineage-specific cells using the EasySep Hematopoietic Progenitor Enrichment Kit (StemCell Technologies) as per manufacturer's recommendations. One million enriched haematopoietic progenitor cells were placed into each well of six-well plates in 2 ml of RPMI supplemented with 10% fetal bovine serum and 20 ng ml^−1^ Granulocyte-macrophage colony-stimulating factor (GM-CSF), Complete medium was replaced every 3 days and cells were collected for further analysis at indicated time points. To assess the effects of tumour-derived factors on DC differentiation, Lin^−^ cells were treated with complete medium supplemented with 25% v/v serum-free medium conditioned overnight by subconfluent cultures of the B16F10 melanoma or control media.

### Plasmids

PGEW-empty and PGEW-Id1 vectors were built from plasmid pCCL.sin.cPPT.PGK.GFP.WPRE as previously described^23^.

### Virus production and titration

Lentiviral vector stocks, pseudotyped with the vesicular stomatitis G-protein were produced by transient co-transfection of 293 T cells and titred on HeLa cells. Viral supernatants were concentrated to titres ≥10^8^ transduction units per ml by ultracentrifugation.

### Transduction of tumour and BM Lin^−^ cells

Lin^−^ cells plated at a density of 1 × 10^6^ cells per ml in StemSpan Serum Free Expansion Medium (StemCell Technologies) were transduced with concentrated virus for 12 h (multiplicities of infection=50–60), washed and resuspended in phosphate-buffered saline (PBS) for transplantation in irradiated mice or subsequent *in vitro* studies.

### Immunofluorescence staining

B16F10 tumours and lung tissues were fixed frozen with Optimal Cutting Temperature compound. Sections (cryostat, Leica) were mounted with Vectashield containing DAPI (4,6-diamidino-2-phenylindole) and were visualized with an ultraviolet fluorescent microscope (Nikon Eclipse E800) with a Retiga camera (QImaging) through IP Lab version 3.65a imaging software (Scanalytics). For GFP and mCherry quantification, only DAPI staining was performed. Using Adobe Photoshop 7.0, × 100 objective fields were analysed by selecting a standardized colour range. After boundary delineation, the area under the pixilation histogram was calculated, comparing total staining area to total tissue area or counting the number of vessels and GFP^+^ cells per field.

### Primers for qPCR

Mouse Id1-forward primer

5′-TTGTTCTCTTCCCACACTCTGTTC-3′

Mouse Id1-reverse primer

5′-CTGGCGACCTTCATGATCCT-3′

Mouse Id1-probe

5′FAM-CAGCCTCCTCCGCTCCCCTCC-3′ TAMRA

All other sets were commercial proprietary Taqman assays purchased form Applied Biosystems.

### OT-I T-cell assays

Equal number of GFP^+^ CD11b^+^ Gr1^+^ cells isolated by FACS from Id1-overexpressing and control vector splenocytes were co-cultured in the presence of OVA_257–264_ peptide with splenocytes isolated from OT-I transgenic mice (C57BL/6-Tg(TcraTcrb)1100Mjb/J, JAX) and stained using CellTrace CFSE Cell Proliferation Kit (Invitrogen). T-cell proliferation was measured by CFSE dilution following a 4-day incubation in 96-well tissue culture-treated plates (Corning).

### OT-II T-cell assays

Single-cell suspensions of splenocytes from Id1-overexpressing and control vector animals (10^5^ cells) were co-cultured in the presence of OVA_323–339_ peptide with 10^5^ CD4^+^ T cells isolated from OT-II transgenic mice (C57BL/6-Tg(TcraTcrb)425Cbn/J, JAX) using the CD4^+^-negative selection kit (Miltenyi Biotec) and stained using CellTrace CFSE Cell Proliferation Kit (Invitrogen). T-cell proliferation was measured by CFSE dye dilution and cytokine production by enzyme-linked immunosorbent assay (ELISA; R&D Systems) following a 72-h incubation in 96-well tissue culture-treated plates (Corning).

### BM transplantation

Recipient mice were lethally irradiated with a single dose of 9.5 Gy of whole-body irradiation. Twenty-four hours after irradiation, 2 × 10^6^ donor lineage-depleted cells isolated from BM cells were injected via tail vein.

### Tumour implantation

C57BL/6 mice were injected in the mammary fat pad with 2 × 10^5^ E0771 cells or intradermally in the flank with 1 × 10^6^ B16F10 cells. Both cell lines were obtained from the American Type Culture Collection. Tumour dimensions were calculated by caliper measurements and volume was calculated according to the equation:


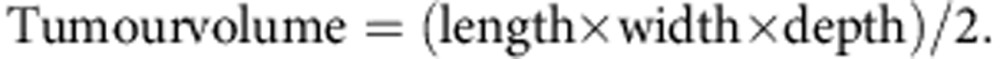


### Cell preparation and flow cytometry

Single-cell suspensions of splenocytes were stained at 4 °C in PBS with 3% (vol/vol) fetal bovine serum, following red blood cell lysis (Gibco, Invitrogen) and incubation with purified Fc-block (CD16/CD32, BD). The following antibodies were used for staining: anti-mouse: anti-CD11c Phycoerythrin (PE) (HL3) (1:100), anti-Gr1 PE (RB6-8C5) (1:50), anti-CD11b Fluorescein isothiocyanate (FITC) (M1/70) (1:100), anti-MHC class II FITC (I-A/I-E; M5/114.15.2) (1:200), anti-major histocompatibility complex (MHC) class II FITC (I-Ek; 14-4-4S) (1:200), anti-CD34 PE (RAM34) (1:100) and anti-IFNγ (XMG1.2) (1:100), all obtained from BD Pharmingen; anti-Ly6G PE (1A8) (1:50) and anti-Ly6C Allophycocyanin (APC) (HK1.4) (1:100), both obtained from Biolegend; anti-CD115 APC (AFS98) (1:100), anti-CD49b PE-Cy7 (DX5) (1:200), anti-CD3 PE-Cy7 (145-2C11) (1:50), anti-CD19 PE-Cy7 (1D3) (1:300), anti-Ter119 PE-Cy7 (TER119) (1:200), anti-Gr1 PE-Cy7 (RB6-8C5) (1:300), anti-CD117 (c-kit) APC-eFluor780 (2B8) (1:200), anti-CD16/CD32 Alexa700 (93) (1:50), anti-Sca-1 PE-Cy5 (D7) (1:200), anti-CD135 biotin (A2F10) (1:200), Streptavidin PerCP-Cy5.5 (1:300), anti-CD4 FITC (RM4-5) (1:200), anti-CD4 Pacific Blue (RM4-5) (1:100), anti-CD25 APC (PC61) (1:100), anti-CD25 Alexa700 (PC61.5) (1:150), anti-Foxp3 PE (FJK-16s) (1:50), anti-CD11b PE-Cy5 (M1/70) (1:200), anti-CD8a APC-eFluor780 (53-6.7) (1:100), anti-V alpha 2 TCR PE (B20.1) (1:200), anti-Gr1 APC (RB6-8C5) (1:200), anti-CD11b APC (M1/70) (1:200) and anti-Gr1 APC-eFluor780 (RB6-8C5) (1:300), and anti-human anti-CD33 PE (WM53) (1:100), CD11B PerCp-Cy5.5 (M1/70) (1:100), anti-CD14 Alexa 700 or FITC (M5E2) (1:100) and anti-HLA PE-Cy7 (L243) (1:200), obtained from BD or eBioscience, and anti-VEGFR1 APC (49560) (1:100) obtained from R&D Systems. Data were acquired on a FACSCalibur, a FACSCanto or an LSR II (BD Biosciences) and analysed with FlowJo software (Treestar). FACS was performed on a Vantage cell sorter (BD Biosciences).

### Measurement of ROS

ROS was measured by labelling cells with the oxidation-sensitive dye dichlorodihydrofluorescein diacetate; (Abcam) according to the manufacturer's instructions, and analysis was carried out by flow cytometry on a FACSCalibur (BD Biosciences).

### qPCR analysis

Total RNA was extracted from cells with RNeasy Mini Kit (Qiagen). Genomic DNA was removed by treatment with DNase I (Qiagen). Complementary DNA (cDNA) was synthesized using the Superscript III reverse transcription kit (Invitrogen). qPCR was performed on a 7,500 Fast Real Time PCR System (Applied Biosystems) using TaqMan Universal PCR Master Mix (Applied Biosystems). Primer assays were purchased from Applied Biosystems or sequences are available in Supplementary Methods. Relative expression was normalized to β-actin levels.

### Microarray preparation and analysis

Total RNA was isolated from Lin^−^ BM cells transduced with Id1-overexpressing or control lentivirus and cultured for 6 days as described above, using the RNeasy Mini Kit (Qiagen). The Affymetrix One-Round *In Vitro* Transcription RNA Amplification Kit was used to amplify 1.5 μg of total RNA. The cDNA was synthesized with a primer containing oligo(dT) and T7 RNA polymerase promoter sequences. Double-stranded cDNA was then purified and used as a template to generate biotinylated complementary RNA (cRNA). The quantity and quality of the amplified cRNA was assessed using a NanoDrop ND-1000 Spectrophotometer (Thermo Scientific) and an Agilent Bioanalyzer. The biotinylated cRNA was fragmented and hybridized to Affymetrix Mouse Genome 430A 2.0 arrays representing ∼14,000 well-characterized mouse genes. After hybridization, the GeneChip arrays were washed, stained and scanned using a GeneChip Scanner 3,000 7G. Affymetrix GeneChip Operating Software was used for image acquisition. Analysis was performed using GeneSpring GX 15.11 software (Agilent Technologies Inc., USA). Robust Multichip Average with Quantile normalization was used for background correction and normalization of CEL files. Genes differentially expressed were identified by using a fold change cutoff of 1.4. Pathway analysis of differentially expressed genes was carried out using IPA to determine significant gene networks and canonical pathways in IPA version 8.6 (Ingenuity Systems, www.ingenuity.com).

### ELISA

Plasma levels of IFNγ, IL-10 and TGFβ were determined using the Mouse IFNγ and IL-10 Quantikine ELISA Kits and Human TGFβ1 Quantikine Elisa (R&D Systems,) according to manufacturer's instructions.

### B16F10 exosome purification

To isolate exosomes, serum-free B16F10-conditioned media was centrifuged at 500*g* for 10 min. The supernatant was then removed and re-centrifuged at 12,000*g* for 20 min. Exosomes were then harvested by centrifugation at 100,000*g* for 70 min. The exosome pellet was resuspended and washed in 20 ml of 1 × PBS and collected by centrifugation at 100,000*g* for 70 min (Beckman Optima XE ultracentrifuge equipped with TY-70Ti rotor). Freshly isolated B16F10 exosomes were added to Lin^−^ cell cultures at 10 μg ml^−1^.

### Western blot

Three million CD11b^+^ splenocytes isolated using CD11b^+^ micro-beads (Miltenyi Biotec) from naive or B16F10-tumour-bearing mice were lysed in 100 μl RIPA cell lysis buffer (Thermo Scientific) containing a cocktail of protease inhibitors (Roche). The supernatant of cell lysis was subjected to western blotting analysis with anti-mouse ID1 (Biocheck, 1:200) and anti-β-actin antibodies (Santa Cruz, 1:100). The western blot was carried out in three independent replicate experiments.

### Immunofluorescence staining

B16F10 tumour tissues and lung tissues were fixed in 4% paraformaldehyde before being embedded in Optimal Cutting Temperature compound. Immunofluorescence staining was performed using rat anti-mouse CD31 antibody (BD Biosciences), biotinylated anti-rat IgG as a secondary antibody and Texas Red Avidin DCS (Vector Laboratories, Inc.). Cryosections (Leica cryostat) were mounted with Vectashield containing DAPI and were visualized with an ultraviolet fluorescent microscope (Nikon Eclipse E800) with a Retiga camera (QImaging) through IPLab version 3.65a imaging software (Scanalytics).

### Statistical analysis

Statistical and graphical analyses were performed using GraphPad Prism software (version 3.0). Data were analysed using Student's unpaired *t*-test, one-way analysis of variance and Mann–Whitney test. Results were considered statistically significant at *P* values <0.05. Error bars depict s.e.m.

## Author contributions

M.P. developed the hypothesis, designed the experimental approach, performed the experimental work, analysed the data, coordinated the project and wrote the manuscript. I.M. conducted experimental work and analysis, contributed to data interpretation and experimental design, and edited the manuscript. Y.H. conducted experimental work and analysis, and contributed to experimental design. M.d.R.A. and H.B.-M. conducted experimental work and analysis, and edited the manuscript. A.S.C., J.K., I.R and Q.W. conducted experimental work and analysis. C.W. processed human blood specimens, managed the mouse colony and conducted experimental work. J.D.W., P.B.C., H.P., A.J.O., R.N.K. and J.B. discussed the hypothesis, contributed to experimental design and obtained human blood specimens. N.A. discussed the hypothesis and edited the manuscript. J.P.G. discussed the hypothesis and contributed to experimental design. D.S. conceived the hypothesis, led the project, interpreted the data and wrote the manuscript. D.L. conceived the hypothesis, led the project, interpreted the data and wrote the manuscript.

## Additional information

**Accession codes:** Microarray data has been deposited in the ArrayExpress database (www.ebi.ac.uk/arrayexpress) under accession code E-MTAB-2280.

**How to cite this article:** Papaspyridonos, M. *et al.* Id1 suppresses anti-tumour immune responses and promotes tumour progression by impairing myeloid cell maturation. *Nat. Commun.* 6:6840 doi: 10.1038/ncomms7840 (2015).

## Supplementary Material

Supplementary Information Supplementary Figures 1-6 and Supplementary Table 1
