# Supplementary material for: Id1 suppresses anti-tumour immune responses and promotes tumour progression by impairing myeloid cell maturation
Source: Nat Commun. 2015 Apr 29;6:6840. doi: 10.1038/ncomms7840 (PMC4423225; doi:10.1038/ncomms7840)
Supplement: Supplementary Information — Supplementary Figures 1-6 and Supplementary Table 1 [file ncomms7840-s1.pdf]

# Supplementary Figures

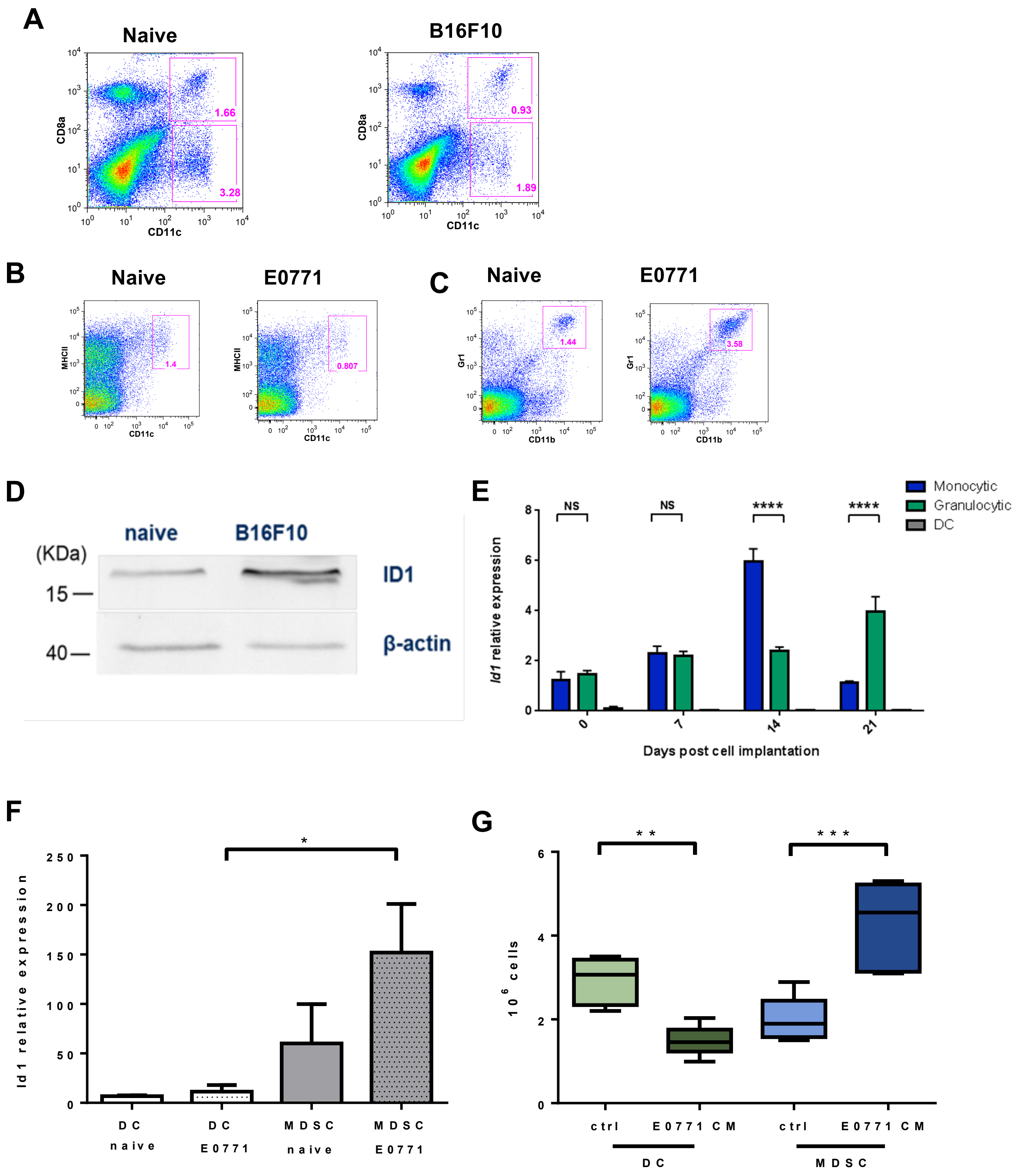

**Supplementary Figure 1. Tumour-secreted factors favour bone marrow-derived cell differentiation towards high Id1-expressing MDSCs but not DC differentiation *in vivo* and *in vitro***

**(**A) Representative frequencies of CD8+ and CD8- DCs by flow cytometry analysis of spleens from naïve and B16F10 melanoma-implanted and control mice (Day 21 post implantation). Representative plots of flow cytometry analysis of spleens from E0771 mammary adenocarcinoma -implanted mice (Day 21 post implantation) for **(B)** DC frequencies and **(C)** MDSC frequencies compared to control mice. **(D)** Id1 protein levels in lysates from naïve and B16F10 bearing CD11b+ bead sorted splenocytes as determined by western blot (Representative results from three independent experiments). **(E)** Id1 mRNA expression levels of FACS-sorted splenic DC (CD11c$^{+}$), monocytic (CD11b$^{+}$Ly6C$^{+}$) and granulocytic (CD11b$^{+}$Ly6G$^{+}$) MDSC populations from spleens from naïve and B16F10 melanoma-bearing mice, on days 7, 14 and 21 following implantation as determined by qPCR analysis (means ± SEM, n = 5, One-way ANOVA, $^{****}p<0.001$). **(F)** Id1 mRNA expression levels of FACS-sorted splenic DC and MDSC populations from spleens from E0771 mammary adenocarcinoma-implanted mice, as determined by qPCR analysis (means ± SEM, n = 6, One-way ANOVA, [illegible]$p < 0.05$). **(G)** *In vitro* differentiation of lin$^{-}$ cells, cultured for 6 days in the presence of E0771 mammary adenocarcinoma TCM (25% v/v) and analyzed for DC and MDSC content by flow cytometry (means ± SEM, n = 6, Unpaired t-tests, $^{**}p < 0.01$, $^{***}p < 0.001$).

## Supplementary Figure 2

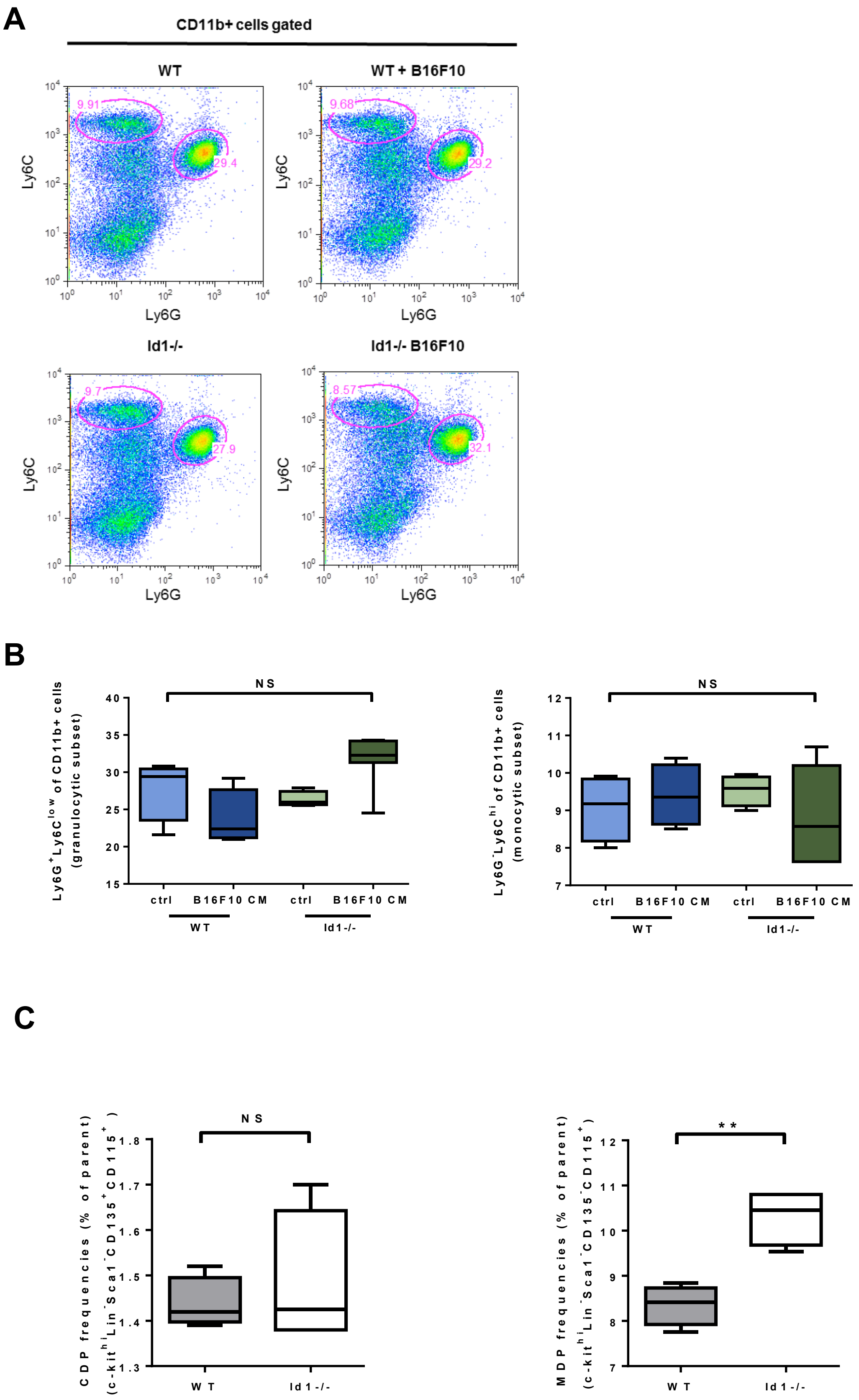

**Supplementary Figure 2. Analysis of MDSC subsets.**

**(A)** Flow cytometry analysis of granulocytic and monocytic MDSC subsets from WT and Id1-/- splenocytes of mice injected daily with B16F10 TCM or control media over 21 days **(B)** Summary graphs of granulocytic and monocytic subset levels across all animal groups (One-way ANOVA, not significant)**. (C)** Flow cytometry analysis of common dendritic cell progenitor (CDP) and myeloid dendritic cell progenitor (MDP) frequencies in bone marrow samples from WT and Id1-/- animals (Unpaired t-tests, NS: not significant, $^{**}p < 0.01$).

**Supplementary Figure 3**

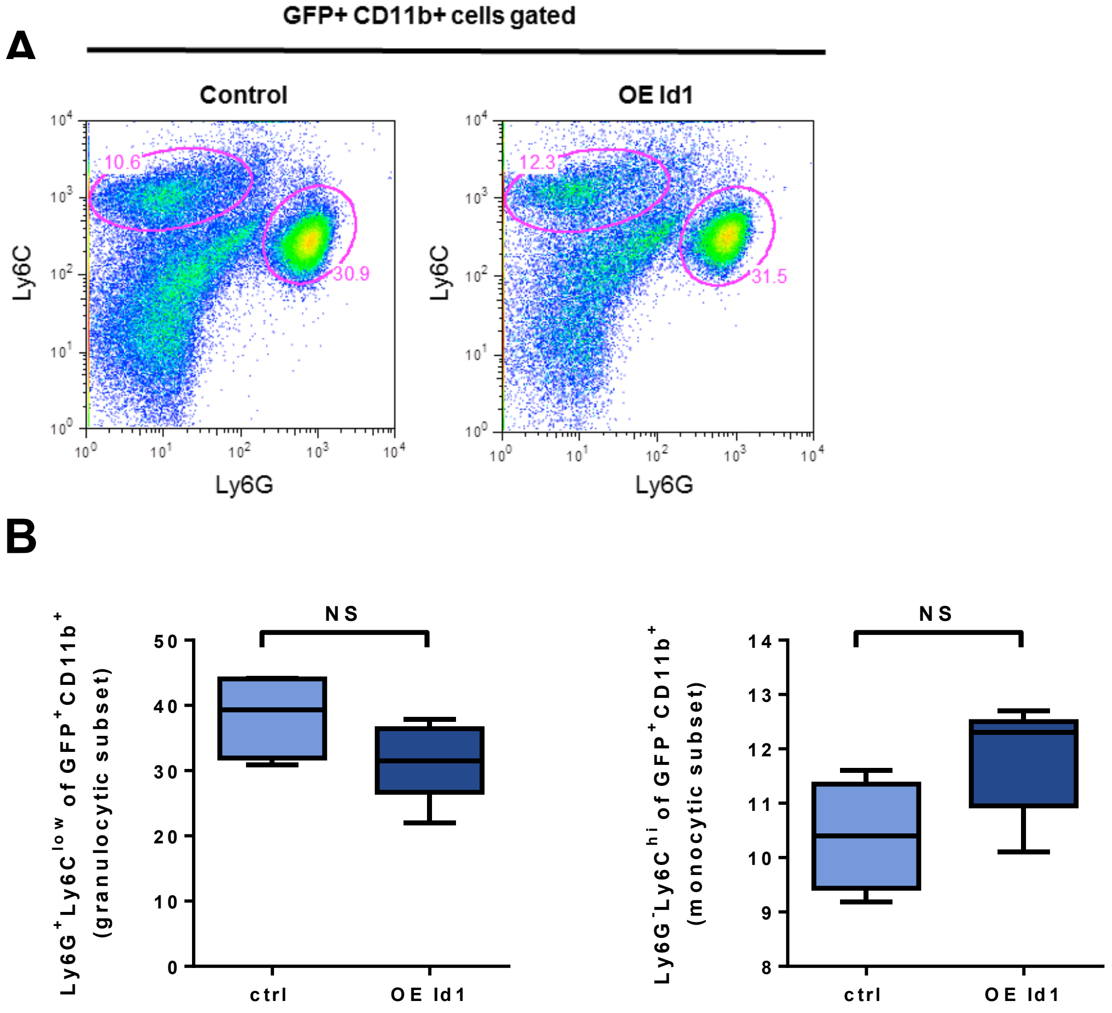


**Supplementary Figure 3. Analysis of MDSC subsets.**

**(A)** Flow cytometry analysis of granulocytic and monocytic MDSC subsets from mice transplanted with control-vector and Id1-overexpressing-vector transduced lin$^{-}$ bone-marrow cells **(B)** Summary graphs of granulocytic and monocytic subset levels (Unpaired t-test, NS: not significant).

**Supplementary Figure 4**

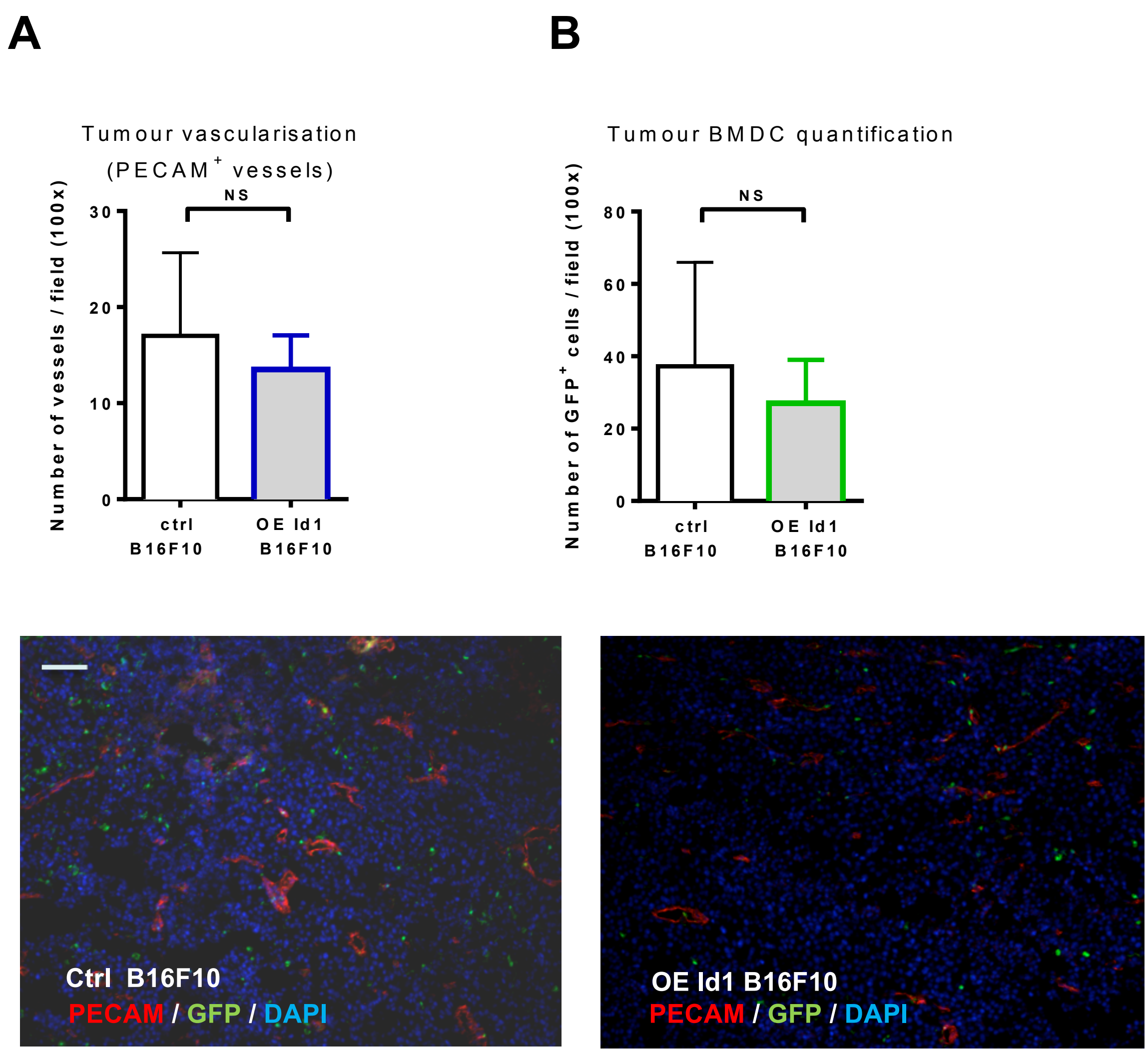


**Supplementary Figure 4. Primary tumour analysis by immunofluorescence (A)** Immunofluorescence analysis of vascularization of platelet/endothelial cell adhesion molecule-1 (PECAM-1) (Texas Red staining quantification) and **(B)** bone marrow cell infiltration (GFP+ quantification) in primary B16F10 tumours from mice transplanted with control-vector and Id1-overexpressing-vector transduced lin$^-$ bone-marrow cells. Scale bar: 50 µm (Unpaired t-tests, NS: not significant).

# Supplementary Figure 5

A

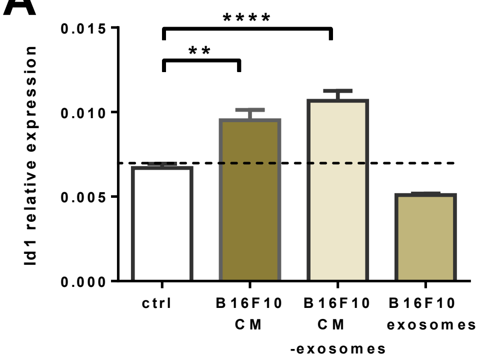


B

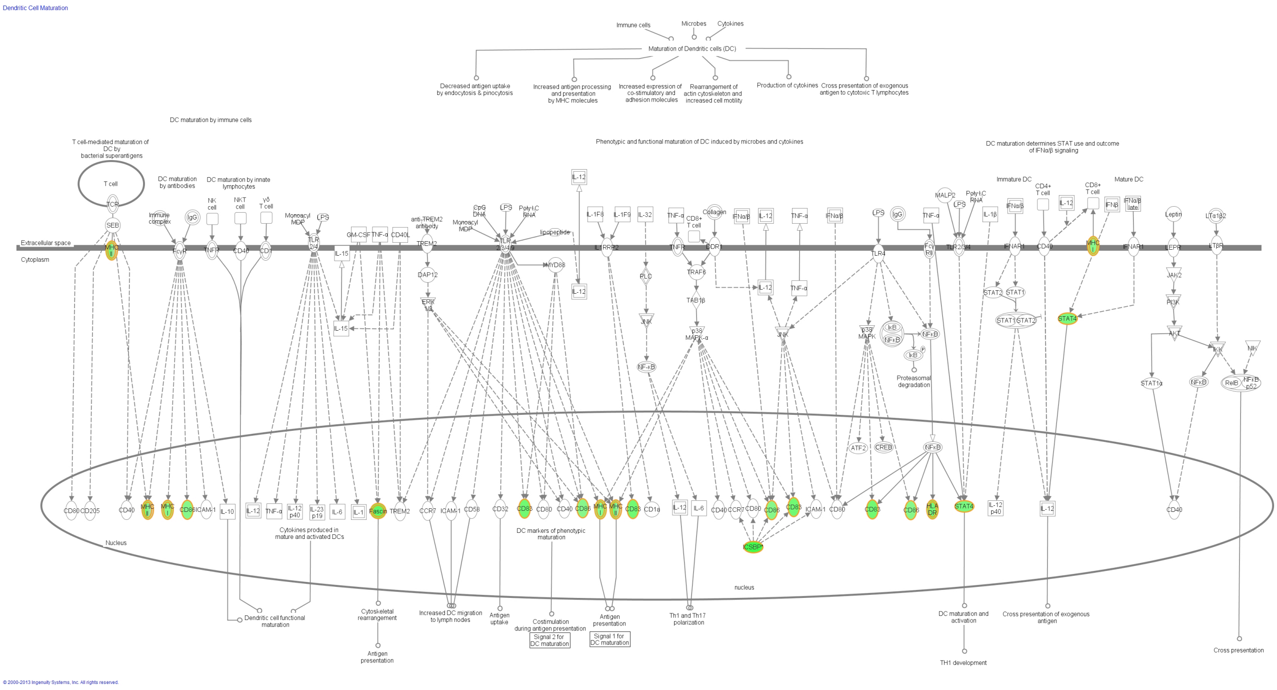


C

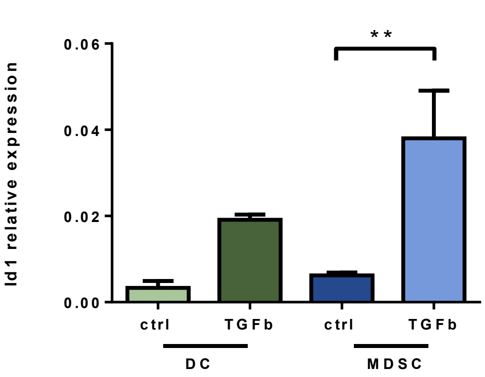


D

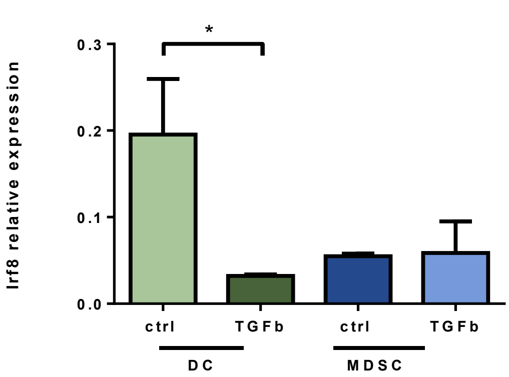

**Supplementary Figure 5.**

**(A)** Id1 relative mRNA expression levels of in vitro differentiation of $lin^-$ hematopoietic progenitors isolated from C57Bl/6 mice, cultured for 6 days in the presence of B16F10 CM, B16F10 CM depleted from exosomes and B16F10 exosomes, as determined by qPCR analysis (means ± SEM, n = 6, One-way ANOVA, **p < 0.001).

**(B)** Id1 downstream mediators. Pathway analysis of differentially expressed genes in Id1 overexpressing BMDC. DC maturation is one of the top pathways affected by Id1 overexpression as determined using Ingenuity Pathway Analysis software. Relevant downregulated genes are shown in green.

**(C)** Id1 and **(D)** Irf8 mRNA expression levels of FACS-sorted DC and MDSC populations following in vitro differentiation of $lin^-$ cells, cultured for 6 days in the presence of recombinant TGFβ, as determined by qPCR analysis (means ± SEM, n = 5, One-way ANOVA, **p < 0.01, *p < 0.05).

## Supplementary Figure 6

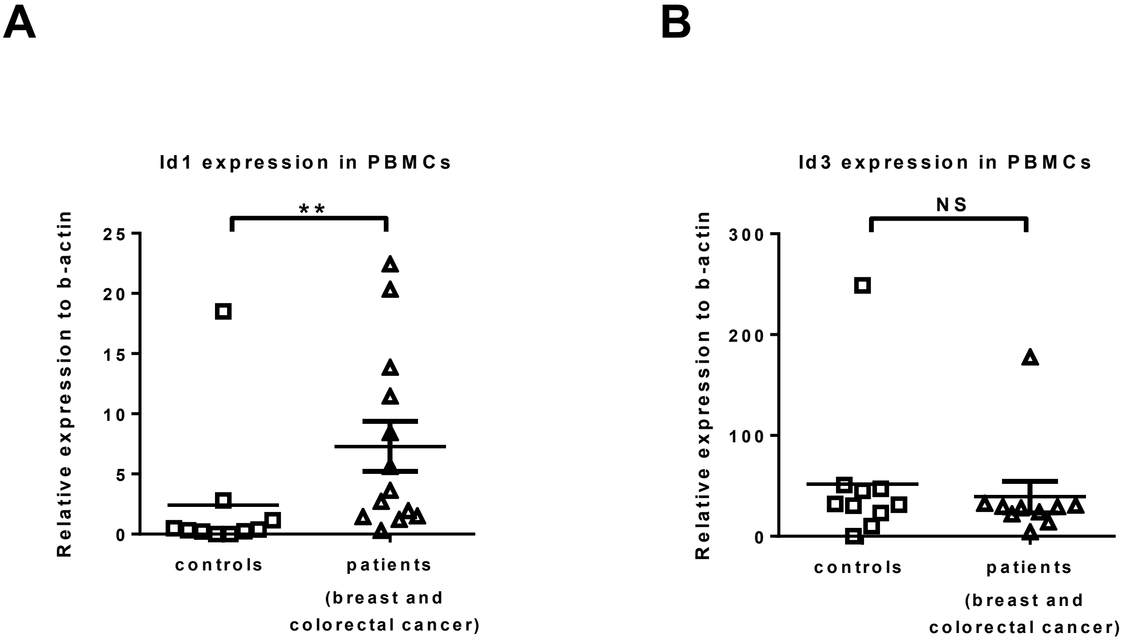


**Supplementary Figure 6. PBMCs from patients with metastatic breast and colorectal cancer express higher levels of Id1 compared with PBMC from controls.**

**(A)** qPCR analysis of Id1 (Mann Whitney test, $^{**}p < 0.01$) and **(B)** Id3 mRNA expression levels following isolation of PBMCs from metastatic colorectal and breast cancer patient blood samples (n=13) compared to healthy age-matched controls (n=10).

**Supplementary table**

**Supplementary Table 1.** DC maturation gene signature. List of DC maturation-associated differentially expressed genes following Id1 overexpression compared to control BMDC

| Symbol | Entrez Gene Name | Affymetrix | Fold Change | Entrez Gene ID for Mouse |
|---|---|---|---|---|
| IRF8 | interferon regulatory factor 8 | 1416714_at | -2.048 | 15900 |
| FSCN1 | fascin homolog 1, actin-bundling protein | 1416514_a_at | -1.786 | 14086 |
| HLA-DQA1 | major histocompatibility complex, class II, DQ alpha 1 | 1452431_s_at | -1.669 | 14960 |
| CD83 | CD83 molecule | 1416111_at | -1.669 | 12522 |
| HLA-DRB1 | major histocompatibility complex, class II, DR beta 1 | 1417025_at | -1.642 | 14969 |
| STAT4 | signal transducer and activator of transcription 4 | 1448713_at | -1.557 | 20849 |
| HLA-DQB1 | major histocompatibility complex, class II, DQ beta 1 | 1451721_a_at | -1.465 | 14961 |
| HLA-A | major histocompatibility complex, class I, A | 1425137_a_at | -1.425 | 15007 |
| CD86 | CD86 molecule | 1420404_at | -1.408 | 12524 |
